# Public hospital reform, family health consumption and health inequality: evidence from China Family Panel Studies

**DOI:** 10.3389/fpubh.2024.1352417

**Published:** 2024-06-18

**Authors:** Lan Jiang, Yiqing He, Chunqi Hu

**Affiliations:** ^1^School of Public Policy and Administration, Nanchang University, Nanchang, China; ^2^Department of Operations and Finance, The First Affiliated Hospital, Nanchang University, Nanchang, China; ^3^School of Economics and Management, Nanchang University, Nanchang, China

**Keywords:** public hospital reform, health inequality, healthcare policies, household consumption structure, China Family Panel Studies

## Abstract

**Background:**

In 2017, China launched a comprehensive reform of public hospitals and eliminated drug markups, aiming to solve the problem of expensive medical treatment and allow poor and low-income people to enjoy basic health opportunities. This study attempts to evaluate the policy impact of public hospital reform on the health inequality of Chinese residents and analyze its micro-level mechanism from the perspective of household consumption structure. Studying the inherent causal connection between public hospital reform and health inequality is of paramount significance for strengthening China’s healthcare policies, system design, raising the average health level of Chinese residents, and achieving the goal of ensuring a healthy life for individuals of all age groups.

**Methods:**

Based on the five waves of data from the China Family Panel Studies (CFPS) conducted in 2012–2020, We incorporates macro-level statistical indicators such as the time of public hospital reforms, health insurance surplus, and aging, generating 121,447 unbalanced panel data covering 27 provinces in China for five periods. This data was used to explore the impact of public hospital reform on health inequality. Logical and empirical tests were conducted to determine whether the reform, by altering family medical care and healthy leisure consumption expenditures, affects the micro-pathways of health inequality improvement. We constructed a two-way fixed model based on the re-centralized influence function (RIF_CI_OLS) and a chained mediation effects model to verify the hypotheses mentioned above.

**Results:**

Public hospital reform can effectively improve the health inequality situation among Chinese residents. The reform significantly reduces household medical expenses, increases healthy leisure consumption, promotes the upgrading of family health consumption structure, and lowers the health inequality index. In terms of indirect effects, the contribution of the increase in healthy leisure consumption is relatively greater.

**Conclusion:**

Public hospital reform significantly alleviates health inequality in China, with household health consumption serving as an effective intermediary pathway in the aforementioned impact. In the dual context of global digitization and exacerbated population aging, enhancing higher education levels and vigorously developing the health industry may be two key factors contributing to this effect.

## 1 Introduction

In April 2023, the World Health Organization initiated the Health Inequality Database, tracking the evolving health disparities among different populations over time. It called on nations to adopt routine monitoring of health inequality, incorporating it into global and national objectives. The health inequality discussed in the academic circle and in this article refer to health opportunity inequality, a concept derived from the theory of equal opportunity (1). This theory divides inequality based on responsible entities into “inequality caused by individually controllable factors” and “inequality resulting from factors beyond individual (2), responsibility, namely, opportunity inequality.” Health opportunity inequality can lead to impoverished and low-income populations being unable to accumulate health capital, perpetually trapped in a cycle of poverty, exacerbating socio-economic inequality. Scholars, after calculating concentration indices for some countries or regions, have found varying degrees of health inequality (3–5). Uneven supply of public resources and socioeconomic status are the main factors influencing health inequality (6). Insufficient total healthcare resources, irrational structural distribution, and a lack of quality resources on the healthcare supply side are significant contributors to unfair medical services and exacerbate health inequality (7). Meanwhile, the essence of socioeconomic status factors is to create unequal access to resources among different groups, thereby affecting the utilization of healthcare services and leading to health inequality (2). Socioeconomic status factors include direct factors such as personal or parental income gap (8), housing (9), pressure (10), education (11, 12), as well as indirect differences such as internet function utilization (13), Self-Efficacy (14), and residency requirements (2).

Health opportunity inequality is susceptible to policy intervention (15). In 2009, China embarked on a new phase of healthcare system reform, implementing a series of measures to continually enhance the distribution of medical resources, reduce the out-of-pocket expenses for residents, and improve the accessibility of healthcare services. The reform has yielded significant results. According to data from the National Health Commission’s Health Development Statistics Bulletin, the preliminary estimate of China’s total health expenditure in 2022 is 84,846.7 billion yuan, accounting for 7.0% of the GDP, representing a 0.5% increase from 2021. Notably, government health expenditure reached 23,916.4 billion yuan, constituting 28.2% of the total health expenditure. In rural areas, the number of practicing (assistant) physicians per thousand people increased to 2.32, narrowing the urban–rural gap from 1.66 people in 2021 to 1.38 people. By the end of 2022, China had established 17 national medical centers across 10 categories, piloting the construction of 26 national regional medical centers, thereby progressively expanding high-quality medical resources to underserved areas. Within the series of healthcare reform policies, the effectiveness of the new rural cooperative medical care policy is unclear and may even contribute to widening wealth disparities (16), and the equalization of basic public health services has narrowed the health gap between the mobile population and the local population, but has exacerbated health inequalities among the mobile population (17). Further refinement of the institutional design is needed in this regard. Conversely, policies such as the integration and coordination of urban and rural residents’ medical insurance; and hierarchical diagnosis and treatment demonstrate a positive impact on promoting health equity (18–21).

As a cornerstone of China’s healthcare system, the comprehensive reform and development of public hospitals are crucial components of healthcare system reforms. In February 2010, the pilot for public hospital reform was initiated, identifying 16 national-level and 37 provincial-level pilot projects to explore reforms in separation of hospital management from operations; separation of medical services from drug sales; separation of hospital from public institutions and separation of for-profit privately-financed from non-profit publicly-financed aspects of hospital operations. In 2017, the comprehensive reform pilot of urban public hospitals was fully implemented. By the end of 2017, 93.9% of urban public hospitals had abolished the drug mark-up. Scholars’ discussions on the effectiveness of public hospital reform mainly revolve around two aspects: medical expenses and the quality of medical services. The Jiangsu Provincial Price Bureau research team compared the data of 14 sample hospitals in Jiangsu Province, China, before and after the reform of public hospitals (2014–2016). They found that the surplus rate of drug income and expenditure in sample hospitals decreased by 16.05%, and the proportion of drug income decreased from 46.59 to 40.52% (22). Chen et al. (23) based on medical insurance reimbursement data from a provincial capital city in China and estimated using a difference-in-differences model, observed that the separation of medicine and medical treatment improved the income structure of public hospitals, but the effect on reducing patients’ medical costs was not significant. Scholar He et al. (24) investigation, employing annual statistical data from medical and health institutions in Sichuan Province spanning 2014 to 2018, indicated that following the division of medicine and medical treatment, there was a short-term reduction in both outpatient and inpatient total costs, yet the long-term alterations were not notable. Wang and Zha (25) based on municipal-level data from 2006 to 2018, used a progressive double difference method and a mediation effect model, concluding that *per capita* fiscal expenditure on medical and health care is an important mechanism for improving the service capacity of medical and health care under the pilot reform. Li et al. (26) believe that the pilot reform mainly improves the quality of medical and health care services by increasing the income of medical and nursing staff and reducing the cost of drug procurement.

Overall, current research on the pathways through which policies affect health inequality among residents mainly focuses on macro-level aspects such as the accessibility of medical services. There have been no studies by scholars on the impact of China’s public hospital reform on health inequality, nor have they explored the pathways through which these effects are generated from the micro perspective of household consumption. Although this reform primarily targets individual medical expenses, it is important to recognize that consumption decisions affecting individual health are not solely individual decisions. On the one hand, family members rely on each other financially. Individual consumption decisions, especially in disease treatment, are influenced by the family’s financial situation (27, 28). Moreover, individual medical expenses may increase family financial vulnerability (29, 30) and reduce the budget available for discretionary consumption (31), thereby affecting the health and quality of life of family members (32). On the other hand, the family is often the core unit for health consumption decisions. These decisions, covering aspects such as travel, purchase of over-the-counter drugs, medical equipment, and fitness equipment, typically prioritize the long-term health and well-being of family members and are based on consideration of the overall health of the family. Selecting indicators of household health consumption allows us to explore the bridging role of such consumption from a more comprehensive and long-term perspective between public hospital reform and individual health inequality. Therefore, investigating whether the comprehensive reform of urban public hospitals contributes to the improvement of health opportunity inequality and understanding how this reform operates at the level of each family unit to influence individual health is essential.

Health consumption refers to the expenditures and purchasing behaviors of individuals or families aimed at maintaining and enhancing their health and well-being. This encompasses preventive, diagnostic, therapeutic, and rehabilitative medical expenditures, as well as various leisure activities that promote physical and mental health (33). We define health consumption upgrade as gradually increasing expenditures on activities such as sports and leisure travel within daily household life, while ensuring a budget for medical expenses related to disease treatment and health restoration. Utilizing data from CFPS and provincial-level data, the author employed the re-centralized influence function model (RIF-CI-OLS) with various robust estimation methods and found that the reform of public hospitals in China effectively mitigates residents’ health inequality indices. Heterogeneity analysis indicates that this impact is particularly pronounced among individuals with higher educational levels and less pronounced aging. The results of the chained mediation effects model indicate that the reform, by altering the structure of health consumption in Chinese households—reducing the proportion of medical expenses and increasing the proportion of expenditures on healthy leisure activities—affects the health inequality situation among residents.

This paper extends previous research by proposing that public hospital reform may influence residents’ health consumption structures, enabling them to derive additional health benefits from increased healthy leisure expenditures, thereby altering their health inequality situations. This micro-level empirical support provides insights for China’s ongoing healthcare reforms, emphasizing the need for multifaceted coordination and increased policy intervention to reduce health opportunity inequality. Moreover, it offers empirical lessons for other countries grappling with policies aimed at improving health inequality. The structure of this study is as follows: Part 2 outlines the data, sample selection process, and research methods. Part 3 presents the research findings. Parts 4 and 5 offer discussions and conclusions.

## 2 Methods

### 2.1 Sample selection and screening

As of the present, Beijing University’s China Social Science Survey Center has released results data from six rounds (conducted every 2 years) of the nationwide “China Family Panel Studies” (CFPS) project. Since the comprehensive reform of urban public hospitals was concentrated between 2014 and the end of 2017, and the household consumption expenditure classifications in CFPS differ between the 2010 round and the subsequent five rounds, this study utilized the CFPS database from the years 2012 to 2020. To construct the necessary dataset, macro-statistical data were matched with micro-survey data. The matching involved embedding the timing of urban public hospital reform, along with macro-level indicators such as health insurance balances and aging, based on the CFPS respondents’ provinces. Macro-level data were sourced from the “China Health and Health Statistics Yearbook” published by the National Bureau of Statistics and the provincial “Statistical Yearbooks.” The health inequality examined in this paper is income-related health inequality. In China, residents under the age of 16 lack full legal capacity, meaning this group cannot independently earn legitimate labor income. Therefore, residents under the age of 16 (excluding those aged 16) were excluded from the data compilation. Additionally, samples with missing key information and outliers were removed^1^. Ultimately, 121,447 valid samples from 27 provinces were retained, forming unbalanced panel data for regression analysis.

### 2.2 Core variable selection and descriptive statistics

#### 2.2.1 Primary dependent variable

The primary dependent variable in this study is health inequality. As Equation (1), the calculation of health inequality involves two categories of indicators: health level and income level. The question “How do you think your health is” in the CFPS is used as a self-assessed health variable to represent the individual’s health level; the selection of individual income level indicators takes into account that personal medical expenditures are generally based on the family as the main budget subject, so the questionnaire’s “Net household income *per capita*” represents.

#### 2.2.2 Mediator/secondary dependent variable

In our analysis, we have included two mediator variables: Family Medical Consumption (MC) and Family Healthy Leisure Consumption (HLC). In each survey period, participants are asked about various household consumption expenditures over the past 12 months, including food, clothing, housing, household equipment and daily necessities, transportation and communication, education and entertainment, healthcare, and other consumable expenditures. The Family Health Leisure Consumption is calculated by summing up the family’s expenditures on ‘healthcare,’ ‘cultural and recreational,’ ‘travel,’ and ‘beauty’.

#### 2.2.3 Independent variable

The key independent variable, denoted as H, is determined based on the comprehensive reform coverage time of public hospitals in each province. This study takes the reform of urban public hospitals as a representative of public hospital reform. Since the reform times vary among cities within the same province, the latest time when a city within each province announced the complete elimination of drug markups in public hospitals is used as the reference point for the province’s public hospital reform (Supplementary Table S1). Additionally, due to the temporal nature of policy effects, the reform time for each province is lagged by 1 year. Comparing this lagged reform time with the time of residents’ interviews, a value of 1 is assigned for interviews conducted after the reform, and 0 otherwise.

#### 2.2.4 Control variables

This article selects control variables from the following three levels: first, demographic characteristics mainly include gender, age, marriage, and household registration; second, family and social status characteristics^2^ include family size, education level, and whether to participate in medical insurance; third, considering that health inequality may be influenced by the level of regional economic development and the density of medical resources, variables for the eastern, central, and western regions were selected (refer to Supplementary Table S2 for the division), with the natural logarithm of the Gross Domestic Product (GDP) of each province serving as a control variable for regional economic development. Additionally, the natural logarithm of the number of health technicians per thousand population in each province was selected as a control variable for medical resource availability. Table 1 summarizes the names and meanings of the variables used in this article. And we reports the descriptive statistical characteristics of the main variables of the sample based on the implementation of the public hospital reform in the individual’s province at the time of the interview, mainly including the mean as well as the standard deviation of the whole sample, the pre-reform sub-sample and the post-reform sub-sample, as shown in Table 2.

**Table 1:** Names and definitions of main variables.

Variables	Variable meaning
RIF_CI	Recentralization impact index of health inequality, which is calculated according to the health status and income level ranking of the sample, and then the health concentration index is recentralized by using the recentralization influence function.
Public Hospital Reform	Coverage of public hospital reform. If it is overridden, the value is 1, and if it is not overridden, the value is 0.
Urban	The value for urban areas is 1 and for rural areas is 0.
Age	Age of the respondents.
Gender	The value for male is 1, for female is 0.
Marital Status	The value is 1 for single; 2 for married; 3 for dating; 4 for divorced; 5 for widowed.
Years of Schooling	Number of years of schooling of the respondents.
Basic Medical Insurance	For those who have basic domestic medical insurance, the value is 1.
Family size	Number of family members.
District	The value is 0 in the eastern region, 1 in the central region, and 2 in the western region.
Lngdp	Logarithmic value of Gross Domestic Product (GDP).
Lntech	Logarithm of the number of medical technicians per 1,000 population.
Ln_bmi_cz	Instrumental variable whose value is the logarithm of the cumulative balance of basic health insurance for urban workers in the previous year.
Mi	For those who have basic domestic medical insurance, the value is 1.

**Table 2:** Statistical description of key variables.

Variables	(1)	(2)	(3)
	Full-sample	Pre-reform	Post-reform
RIF_CI	0	0	0
	(0)	(0)	(0)
Public Hospital Reform	0.24	–	–
	(0.42)	–	–
Urban	0.47	0.45	0.52
	(0.5)	(0.5)	(0.5)
Age	46.48	46.36	46.86
	(16.71)	(16.71)	(16.68)
Gender	0.49	0.49	0.5
	(0.5)	(0.5)	(0.5)
Marital Status	2.08	2.08	2.06
	(0.84)	(0.84)	(0.83)
Years of Schooling	7.2	6.95	8
	(4.95)	(4.92)	(4.96)
Basic Medical Insurance	0.9	0.9	0.9
	(0.3)	(0.3)	(0.3)
Family size	4.29	4.33	4.22
	(1.96)	(1.94)	(2.02)
District	0.87	0.88	0.84
	(0.82)	(0.83)	(0.82)
Lngdp	9.97	9.87	10.32
	(0.74)	(0.72)	(0.71)
Lntech	1.77	1.71	1.96
	(0.18)	(0.16)	(0.12)
Ln_bmi_cz	15.3	15.07	16.05
	(0.92)	(0.84)	(0.74)
Mi	0.13	0.12	0.15
	(0.34)	(0.33)	(0.35)
N	121447	92786	28661

### 2.3 Measurement methods and modeling

#### 2.3.1 Measurement of health inequality

Currently, there are two main methods for measuring health inequality: the Relative Deprivation Index DI and the Concentration Index CI. In this case, CI is employed to calculate the degree of health inequality among residents, determined by the joint distribution $\;\left(F_{H,F_{Y}}\right)$ of health level $h_{i}$ and income level $R_{i}$ . The calculation expression for CI is:


(1) $$CI=2\mathrm{cov}\left(h_{i},R_{i}\right)/\bar{h}=\frac{1}{n}\overset{n}{\underset{i=1}{\sum }}\left\{h_{i}\left(2R_{i}-1\right)/\bar{h}\right\}$$

In the joint distribution $\left(F_{H,F_{Y}}\right)$ and the Equation (1), $h_{i}$ and random variable H represents the health level of individual i , with $\bar{h}$ representing the average health level of the population. $F_{Y}$ is the ranking function for resident income $y_{i}$ . Each resident’s income rank ratio is denoted by the character $R_{i}$ , representing individual i’s income rank within the population. The health indicator $h_{i}$ can be a positive indicator (e.g., self-rated health) or a negative indicator (e.g., days hospitalized per year). The CI values range from (n−1)/n to (1−n)/n in a closed interval, where n is the population size.

#### 2.3.2 Two-way fixed effects regression model of RIF–CI−OLS for health inequality

The Influence Function (IF) is a statistical tool used to analyze the robustness of distributional statistics, functionals, or data under small perturbations (34). It can be interpreted as the rate of change in the estimated impact on the distributional statistic $v\left(F_{Y}\right)$ for each added observation $y_{i}\;$ (individual income). The Recentered Influence Function (RIF), building upon the IF function, focuses more on the approximate value of the distributional statistic $v\left(F_{Y}\right)$ after removing or altering specific observations $y_{i}$ in the data. The Expressions as in Equation (2):


(2) $$RIF\left\{y_{i},v\left(F_{Y}\right)\right\}=v\left(F_{Y}\right)+IF\left\{y_{i},v\left(F_{Y}\right)\right\}$$

According to the study by Firpo et al. (35), the IF function and RIF function exhibit the following characteristics as in Equation (3) and Equation (4):


(3) $$\int IF\left\{y,v\left(F_{Y}\right)\right\}dF_{Y}=0$$

(4) $$\int RIF\left\{y,v\left(F_{Y}\right)\right\}dF_{Y=}v\left(F_{Y}\right)$$

Here, $v\left(F_{y}\right)$ represents a statistic of the cumulative distribution function $F_{Y}$ . The expected value of *IF* is 0, and the unconditional expectation of the RIF function is the statistic itself. In this study, $v\left(F_{y}\right)$ is the concentration index $v^{CI}$ . The Recentered Influence Function regression model (RIF-OLS) popularized by Firpo et al. (35)which combines the Recentered Influence Function with the Ordinary Least Square to study the impact of changes in covariates on the unconditional distribution of a given outcome variable. The RIF-OLS model uses $RIF\left\{y_{i},v\left(F_{y}\right)\right\}$ as the dependent variable for each observation $y_{i}$ in the data and regresses it on all relevant variables $X^{\prime }$ (3). When combined with the concentration index statistic $v^{CI}$ , it results in the Recentered Influence Function regression model (RIF-CI-OLS) for the health concentration index:


(5) $$RIF\left\{y,v^{CI}\right\}=X^{\prime }\theta +\varepsilon_{i},E\left(\varepsilon_{i}\right)=0$$

Taking the unconditional expectation on both sides of Equation (5) at the same time, combined with the characteristics of the RIF function in Equation (4), we can get the Equation (6):


(6) $$v^{CI}=E\left\{RIF\left(y,v^{CI}\right)\right\}=E\left(X^{\prime }\theta \right)=\bar{X^{\prime }}\theta$$

The coefficient θ represents the marginal impact of the independent variable’s marginal changes on the concentration index $v^{CI}$ . This paper constructs the RIF-CI-OLS regression model, establishing the association between the health inequality index (population level) and explanatory variables (individual level) through RIF (19). This enables the accurate identification of the marginal impact effects of urban public hospital reforms on health equity. The estimated model is:


$$\begin{array}{l} \mathrm{Model}\;1:RIF\left\{y,v^{CI}\right\}=RIF\_CI_{ipt}=\alpha_{0}+\alpha_{1}H\\ \;\;\;\;\;\;\;\;\;\;\;\;\;\;\;\;\;\;\;\;\;\;\;\;\;\;\;\;\;\;\;\;\;\;\;\;\;\;\;\;\;\;\;\;\;\;\;\;\;\;\;\;\;+\alpha_{2}Z_{ipt}+\delta_{i}+\theta_{p}+\varepsilon_{ipt} \end{array}$$

The dependent variable, $\;RIF\_CI_{ipt}$ , represents the health inequality index possessed by individual i residing in province p in year t . $\alpha_{0}$ is a constant. The key explanatory variable H represents the coverage of the public hospital reform policy among the survey respondents. $\;\alpha_{1}$ is the core parameter to be estimated, indicating the impact of implementing public hospital reform on residents’ health inequality. $Z_{ipt}$ represents the control variables. Considering potential significant differences in the environment and residents’ health habits among different provinces, we further control for the fixed effects of the survey year $\delta_{t}$ and the province of the survey respondent $\theta_{p}$ to absorb some variables that do not vary with time and region. $\varepsilon_{ipt}$ represents the random disturbance term, and the direction and significance of the estimated coefficient $\alpha_{1}$ are the focal points of the analysis.

#### 2.3.3 Discussion of endogeneity and robustness

The goal of public hospital reform is to effectively alleviate the issues of expensive and difficult healthcare access for the public. The challenges of difficult and expensive healthcare reflect the increasing and diverse medical needs of the population and the conflicts arising from the insufficient total healthcare resources, unreasonable distribution, and scarcity of high-quality resources in China. Health inequality is a consequence of the difficulties and high costs of seeking medical care, rather than the cause of these phenomena. Therefore, there is no issue of mutual causation between public hospital reform and health inequality.

The causal identification of the effects generated by public hospital reform in this study is mainly challenged by the omission of important explanatory variables. Behind public hospital reform, there may be other unobservable factors, indicating the possibility of omitted variable bias. We employs the instrumental variable method to address this issue and follows the approach of Jiang et al. (36). By reviewing policy documents and examining the factors considered during policy formulation, instrumental variables are selected. The national guidelines (37) emphasize the need for careful calculation when formulating reform plans within the affordable range of local medical insurance funds. Considering the availability of comprehensive data, the author selected the cumulative surplus of the previous year’s urban employee basic medical insurance as the instrumental variable for the urban public hospital reform policy and conducted instrumental variable regression.

The comprehensive reform plans for urban public hospitals are formulated by various cities. After the reform, compensation for public hospitals shifts from three channels—service fees, drug markups, and government subsidies—to two channels—service fees and government subsidies. Changes in drug expenses and medical service fees affect the reimbursement ratio and scope of medical insurance, thereby influencing local medical insurance funds. Therefore, the cumulative surplus of local medical insurance funds from the previous year will impact the formulation and implementation timing of public hospital reform plans in the province or city for the current year. We believe that this instrumental variable meets the requirement for relevance with the core independent variable.

Regarding the exogeneity of the instrumental variable, firstly, the cumulative surplus of basic medical insurance funds in each province is not a direct factor analyzed in the literature affecting health inequality. Secondly, considering that the cumulative surplus of basic medical insurance funds may also influence health equity by affecting the formulation of medical insurance policies and expanding the coverage of basic medical insurance, we regress whether residents participate in urban employee basic medical insurance as the explanatory variable against the balance of urban employee basic medical insurance in the CFPS database. The regression results show no significance, proving that this instrumental variable does not affect health inequality through other pathways.

Additionally, we conducted four types of tests to enhance the robustness of our findings: (1) replacing core explanatory variables, (2) incorporating county-level fixed effects, (3) excluding provinces with excessively low or high sample sizes, and (4) generating mixed cross-sectional data regression. Due to limitations in the instrumental variable and heterogeneity analysis, the baseline regression used the time when each province’s latest announcement of the full cancelation of public hospital drug markups was made in cities. To avoid endogeneity issues arising from measurement errors, the author re-matched the reform time at the prefecture level with the respondents’ interview time, creating a prefecture-level public hospital reform coverage variable. As the timing of county-level public hospital reform overlaps with that of urban public hospital reform, and the main regression model uses the effect of urban public hospital reform to represent the overall public hospital reform effect, county-level fixed effects are introduced on top of time and province double fixed effects to eliminate the impact of county-level public hospital reform. Furthermore, the study considers sampling errors and potential biases in data type selection. In cases of uneven sampling across different regions leading to biased estimation results (38), we excluded provinces with excessively high or low numbers of respondents from the sample and conducted the regression analysis again. Regarding sample data types, following the practices of other scholars, we categorized the sample into experimental and control groups based on whether they were covered by public hospital reform. This resulted in a mixed cross-sectional dataset with 28,661 samples in the experimental group and 92,786 samples in the control group. Group-based calculations were performed for the sample’s health inequality concentration index.

#### 2.3.4 Mediation chain model

**Figure 1:**
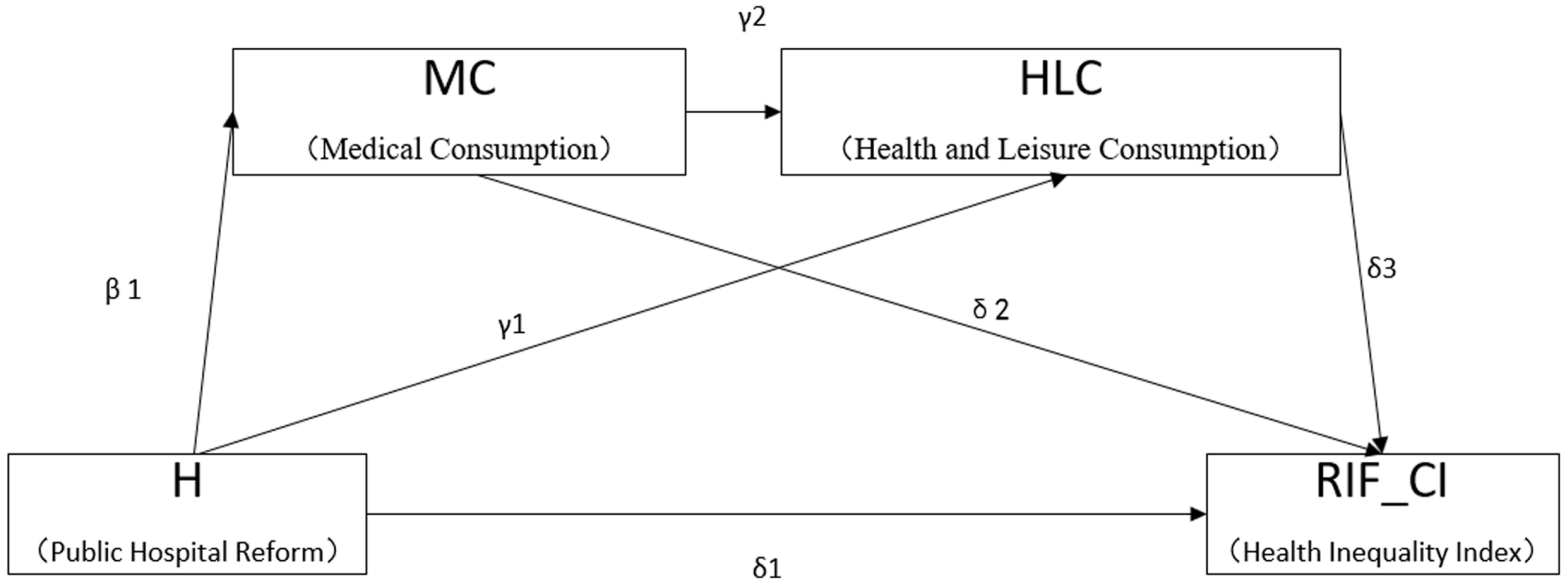
A chain mediation model on the impact of public hospital reform on health inequality.

Based on Assumption 2, the reform, by eliminating drug markups as compensation for public hospitals, reduces residents’ family medical consumption, leading to an increase in the budget for healthy leisure consumption. Since healthy leisure consumption has a mechanism of health reproduction for residents, the increase in this budget effectively narrows the health gap between ordinary households and high-income households, and is reducing the health inequality index. This study constructs a mediation chain model (Figure 1) to examine the main channels through which public hospital reform affects health inequality. The specific model settings are as follows:


$$\mathrm{Model}\;2:MC_{ipt}=\beta_{0}+\beta_{1}H+\beta_{2}Z_{ipt}+\delta_{i}+\theta_{p}+\varepsilon_{ipt}$$

$$\mathrm{Model}\;3:HLC_{ipt}=\gamma_{0}+\gamma_{1}H+\gamma_{2}MC+\gamma_{3}Z_{ipt}+\delta_{i}+\theta_{p}+\varepsilon_{ipt}$$

$$\begin{array}{l} \mathrm{Model}\;4:RIF_{CI}{ip}_{t}=\delta_{0}+\delta_{1}H+\delta_{2}MC+\delta_{3}HLC\\ \;\;\;\;\;\;\;\;\;\;\;\;\;\;\;\;\;\;\;\;\;\;\;\;\;\;\;\;\;\;\;\;\;\;\;\;\;\;\;\;\;\;\;\;\;+\delta_{4}Z_{ipt}+\delta_{i}+\theta_{p}+\varepsilon_{ipt.} \end{array}$$

For residents sampled in the survey year with no family medical expenses, public hospital reform does not impact their health inequality index by reducing the proportion of family medical expenses. Therefore, the mediation regression excludes 12,762 samples with zero family medical expenses. At the same time, to eliminate the dimensional influence among indicators, we standardized the data of family medical expenses and health consumption. The independent mediating effect paths are “H-MC-RIF_CI” and “H-HLC-RIF_CI,” which are recorded as independent mediating effect (1) and independent mediating effect (2). The effect values are $\beta_{1}\delta_{2}$ and $\gamma_{1}\delta_{3}$ respectively. The chain mediation effect path is “H-MC-HLC-RIF_CI”, and the effect value is $\beta_{1}\gamma_{2}\delta_{3}$ . The mediation effect test method (39) is used to test the multiple chain mediation effects. If both β₁ and δ₂ or γ₁ and δ₃ are significant, the independent mediation effects are considered significant. If β₁, γ₂, and δ₃ are all significant, the chained mediation effects are considered significant. If at least one of the above is not significant, the coefficient product is tested using the Bootstrap method.

#### 2.3.5 Discussion on heterogeneity

This study conducts heterogeneity analysis at two levels: residents’ years of education and regional population structure, exploring the differentiated effects of public hospital reform on mitigating health inequality. The informatization reform of public hospitals empowers their high-quality development, and individuals with higher educational levels are more likely to utilize new models such as internet hospitals and medical alliances, improving the efficiency of medical resource utilization and enhancing the accessibility of healthcare services. We followed the approach of Zheng et al. (40) and grouped the sample based on educational levels, dividing them into a lower-educated group (below junior high school) and a higher-educated group (junior high school and above). On the other hand, given the close relationship between population age structure and resident consumption, does the impact of public hospital reform on health inequality vary depending on different population structures? To explore this, the sample data were divided into two groups based on the degree of aging, and regressions were conducted separately. Since aging (population structure) is a dynamic indicator, following the methods of Shen et al. (41) and Chen and Han (42), we used the median degree of aging in each province for each sample year as the basis for grouping.

Data for this study was analyzed using STATA version 17 and statistical significance for all analyses was set at *p* < 0.05.

## 3 Results

Table 3 reports the estimation results of the RIF–CI-OLS regression and instrumental variables regression. Controlling for individual and provincial characteristics, we gradually introduce control variables to examine the policy effects of urban public hospital reform on health inequality in China. The first column of Table 3 controls for survey year fixed effects and provincial fixed effects. The regression results show that the implementation of public hospital reform has a significantly negative impact on the health inequality situation. After the reform, the health inequality index decreases by 0.205 standard deviations at a significant level of 0.01. Columns 2–4 of Table 3 progressively add demographic variables, family and social status variables, and regional variables to the regression. The estimated coefficients of the core explanatory variables in each column show little change and are statistically significant at the 1% level. This suggests a clear negative correlation between the implementation of urban public hospital reform and the health inequality index.

**Table 3:** Baseline regression results.

	(1)	(2)	(3)	(4)	(5)	(6)
Variables	RIF_CI	RIF_CI	RIF_CI	RIF_CI	RIF_CI (IV)	Mi
Public Hospital Reform	−0.205^**^	−0.196^**^	−0.194^**^	−0.203^**^	−1.42e-12^**^	
	(−15.00)	(−14.49)	(−14.57)	(−15.03)	(−25.23)	
Ln_bmi_cz (IV)						−0.012
						(−1.23)
Urban		−0.309^**^	−0.219^**^	−0.219^**^	−1.30e-14^**^	0.005
		(−41.14)	(−29.58)	(−29.61)	(−6.18)	(0.99)
Age		0.001^**^	−0.001^**^	−0.001^**^	−5.16e-16^**^	0.006^**^
		(6.05)	(−4.21)	(−4.29)	(−6.95)	(4.57)
Gender		−0.038^**^	0.012	0.012	4.16e-15	−0.007
		(−5.40)	(1.70)	(1.74)	(2.22)	(−0.29)
Marital Status		0.007	−0.001	−0.001	4.18e-15^*^	0.005^*^
		(1.50)	(−0.30)	(−0.30)	(3.14)	(2.16)
Years of Schooling			−0.030^**^	−0.030^**^	−1.92e-15^**^	0.003^**^
			(−35.24)	(−35.25)	(−7.92)	(4.49)
Basic Medical Insurance			−0.054^**^	−0.054^**^	−1.22e-14^**^	
			(−4.83)	(−4.84)	(−3.39)	
Family size			0.059^**^	0.058^**^	9.66e-15^**^	−0.001
			(32.31)	(32.27)	(18.00)	(−1.92)
District				−0.795^**^	3.38e-13^**^	−0.106
				(−4.33)	(3.83)	(−1.73)
Lngdp				0.147^**^	9.16e-13^**^	0.001
				(2.72)	(23.63)	(0.04)
Lntech				−0.657^**^	−8.58e-13^**^	−0.007
				(−8.36)	(−19.69)	(−0.33)
Constant	−0.112^**^	−0.032^**^	0.051^*^	0.441		0.066
	(−23.66)	(−2.60)	(2.42)	(0.85)		(0.34)
Observations	121,447	121,447	121,447	121,447		121,447
R-squared	0.340	0.352	0.367	0.368		0.007
Year FE	YES	YES	YES	YES	YES	YES
Provcd FE	YES	YES	YES	YES	YES	YES
First-stage regression results						
Ln_bmi_cz (IV)					0.206^**^ (36.14)	
Weak Identification Test (F)					1305.94 {16.38}	
Underidentification Test					1142.20 [0.0000]	

Ps1. Clustered Individual-Level Standard Errors in Parentheses ^**^p < 0.01, ^*^p < 0.05; 2. The curly braces contain the critical value at the 10% level provided by Stock and Yogo (43), and the square brackets contain *P*-value of the Kleibergen-Paap rk LM statistic; 3. The *R*-squared in the second-stage regression results for the instrumental variable regression is negative, so it has not been reported in the table.

Our study employs the cumulative balance of the previous year’s basic medical insurance as the instrumental variable for the urban public hospital reform policy. In the first stage, the regression coefficient of the cumulative balance of the previous year’s basic medical insurance on public hospital reform is significant at the 1% level, with a weak instrumental variable test *F*-value of 1305.94, far exceeding the critical value provided by Stock et al. (43) at the 10% level. At the same time, the Kleibergen-Paap rk LM test *p*-value is 0, passing both the weak identification test and the underidentification test. The results of the second-stage regression show that after replacing the core explanatory variable with the instrumental variable, the sign and significance of the regression coefficient remain unchanged, preliminarily proving the effectiveness of selecting the cumulative surplus of the previous year’s urban employee basic medical insurance as the instrumental variable for public hospital reform. However, as a suitable alternative instrumental variable for public hospital reform, this variable must not only explain the differences in public hospital reform across provinces but also meet the requirements of exogeneity. The data sample type in this study is an unbalanced panel. When using time and province double fixed effects along with cluster clustering, there are cases where the sample size of certain units is too small, resulting in the estimation process not being full rank, and the overidentification test not reported. Therefore, only a single instrumental variable was used to explain public hospital reform in this case and it cannot verify the exogeneity of the instrumental variable through the “overidentification assumption.” Following the approach of Fang and Zhao (44), potential path variables are used for instrumental variable regression. If the coefficient of the regression is not significant, it indicates that the instrumental variable does not change the health inequality index through that path. Using the “whether residents participate in urban employee basic medical insurance” in the CFPS database as the explanatory variable and the logarithm of the cumulative surplus of the previous year’s urban employee basic medical insurance as the explained variable for regression, the results in the sixth column of Table 3 show that the impact is not significant. In columns 3–4 of Table 3, when estimating the mitigating effect of public hospital reform on health inequality, we also include “whether residents participate in basic medical insurance” as a control variable and still find that the effect of the reform is consistently significant. This further validates the exogeneity of the instrumental variable.

Table 4 further reports the regression results of robustness tests. After matching the reform time at the municipal level, the regression coefficient of the reform on residents’ health inequality is −0.216, and it is significant at the 1% level, consistent with the main regression results. In the second column, adding county-level fixed effects does not significantly change the coefficient ( $\alpha_{1}\;$ = −0.203) and significance (*p* < 0.01) of the regression. In column (3), after deleting data from provinces with the most and least samples, the sample size is 104,811, and the regression coefficient is −0.26, but it does not affect the sign and significance of the regression (*p* < 0.01).

**Table 4:** Robustness test of benchmark regression.

	(1)	(2)	(3)
Variables	RIF_CI	RIF_CI	RIF_CI
Public Hospital Reform (province)		−0.203^**^	−0.260^***^
		(−15.07)	(−4.95)
Public Hospital Reform (city)	−0.216^**^		
	(−17.24)		
Observations	121,447	121,447	104,811
*R*-squared	0.368	0.3694	0.569
Year FE	YES	YES	YES
Provcd FE	YES	YES	YES
County FE		YES	

PS: Clustered individual-level standard errors in parentheses ^**^p < 0.01, ^*^p < 0.05.

**Figure 2:**
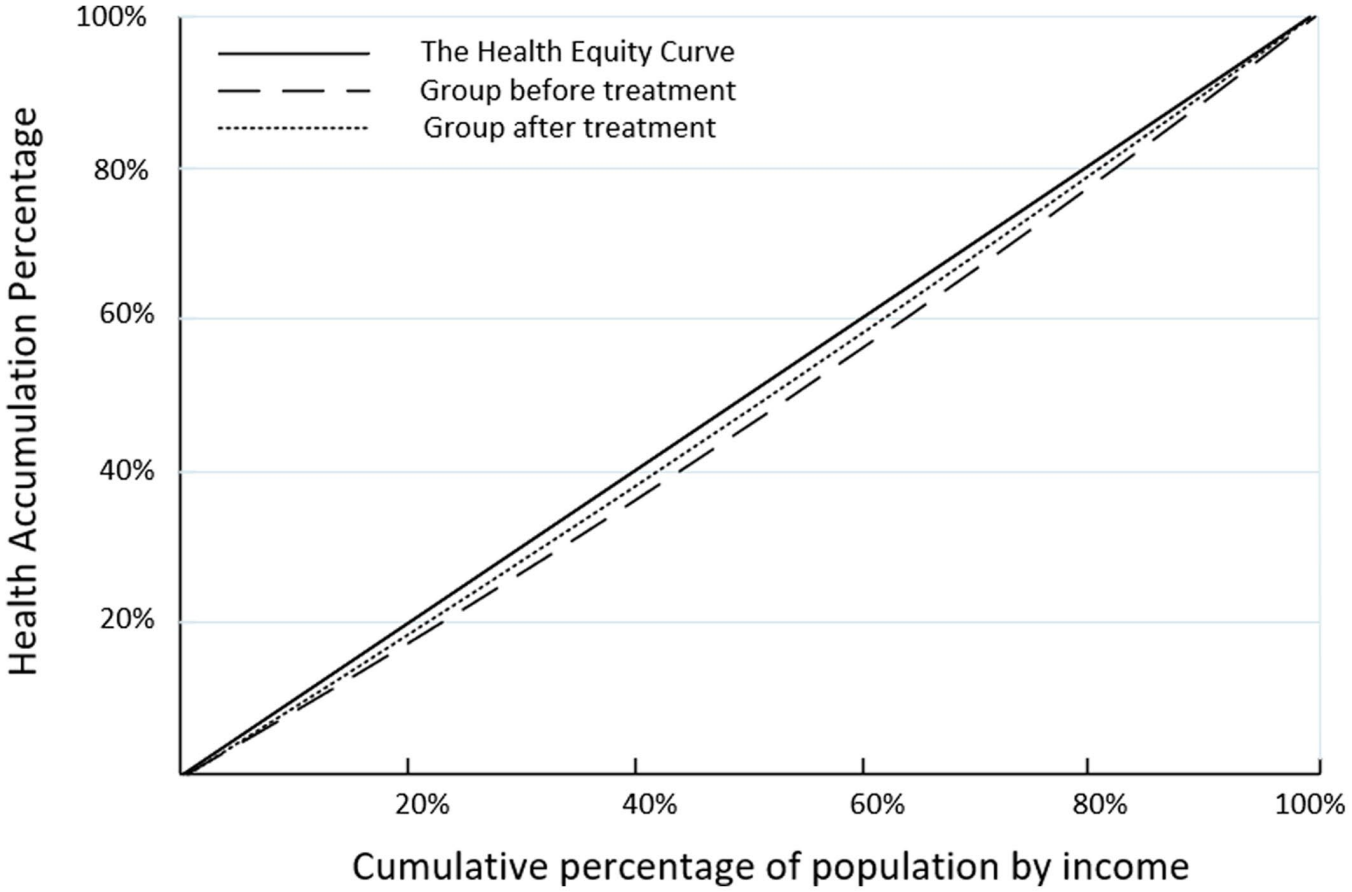
Concentration curve of health inequality among Chinese residents before and after public hospital reform.

Using this sample to calculate CI, the CI for the control group is 0.0153, and for the treatment group, it is 0.0118, showing a decrease of 22.87 percentage points before and after the reform. The health concentration curves for the control group and the treatment group are shown in Figure 2, indicating an improvement in health inequality in China after the separation of medical services from drug sales. The values of CI are greater than 0 before and after the reform, indicating a health inequality that favors the rich among Chinese residents. However, with the progress of the reform, the health disparities favoring higher-income groups are narrowing. In conclusion, the findings of the baseline regression are further strengthened, and research hypothesis 1 is once again confirmed.

**Figure 3:**
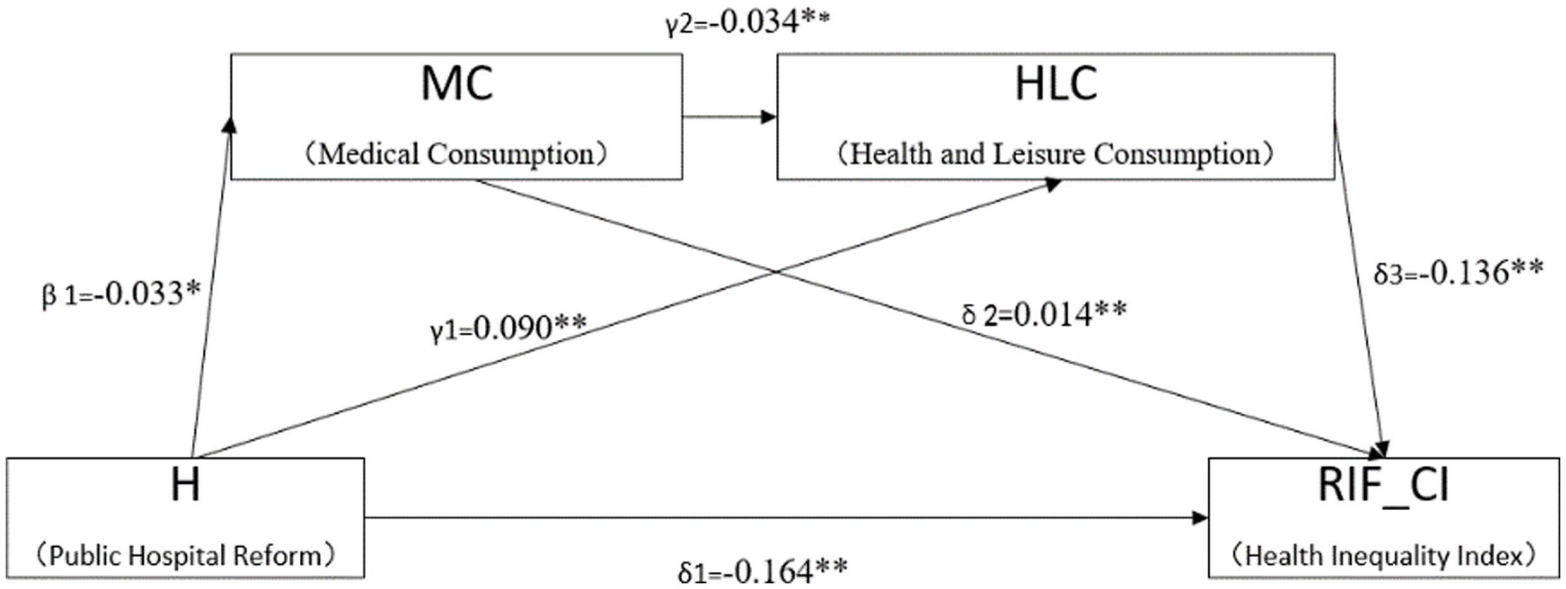
Regression coefficients of the chain mediation model.

The regression results for the chained mediation effects are presented in Table 5. In column (1), it is observed that public hospital reform contributes to a reduction in family medical expenses ( $\beta_{1}$ = − 0.033, *p* < 0.05). In column (2), the coefficients for the impact of public hospital reform (H) and household medical consumption (MC) on family leisure health consumption (HLC) are 0.09 and −0.034, respectively, both significant at the 1% level. This suggests that both medical reform and the decrease in household medical consumption significantly lead to an increase in family leisure health consumption. Column (3) indicates that the upgrading of the structure of family health consumption has a positive effect on reducing residents’ health inequality ( $\delta_{1}$ = −0.164, *p* < 0.01). The effect value of independent mediation 1 ( $\beta_{1}\delta_{2}$ ) is $-4.62*10^{-4}$ , and for independent mediation 2 ( $\gamma_{1}\delta_{3}$ ), it is $-1.22*10^{-2}$ . The chained mediation effect value of public hospital reform on health inequality ( $\beta_{1}\delta_{2}\delta_{3}$ ) is $-1.53*10^{-4}$ . Figure 3 represents the path diagram (including significance levels) illustrating the effect of chain mediation, with clustered individual level standard errors **p < 0.01, *p < 0.05.

**Table 5:** Multiple chain mediation effect regression results.

Chain conduction path: PHR-MC-HLC-RIF_CI			
	(1)	(2)	(3)
Variables	MCR	HLC	RIF_CI
Public Hospital Reform	−0.033^*^	0.090^**^	−0.164^**^
	(−2.19)	(6.42)	(−12.20)
MC		−0.034^**^	0.014^**^
		(−4.09)	(3.02)
HLC			−0.136^**^
			(−17.43)
Constant	1.376^*^	−2.679^**^	−0.785
	(1.81)	(−4.06)	(−1.39)
Control	YES	YES	YES
Observations	108,685	108,685	108,685
Year FE	YES	YES	YES
Provcd FE	YES	YES	YES

PS: Clustered individual-level standard errors in parentheses ^**^p < 0.01, ^*^p < 0.05.

The results of the heterogeneity analysis, as shown in Tables 6A,B, indicate that regardless of whether the educational level is higher or lower, public hospital reform can improve the health inequality status of the target groups. However, the coefficient for the level of improvement among the higher-educated group ( $\alpha_{1}$ = −1.199, *p* < 0.01) is greater than that of the lower-educated group ( $\alpha_{1}$ = −0.530, *p* < 0.01). In regions with a lower degree of aging, the coefficient for the impact of public hospital reform on health inequality is −0.088 at the 1% significance level. However, in areas with a higher degree of aging, this coefficient is not statistically significant.

**Table 6:** Analysis of heterogeneity in years of education and population structure.

Variables	Dependent variable: RIF_CI			
	A		B	
	Higher levels of Eduy	Lower levels of Eduy	Higher levels of aging	Lower levels of aging
Public hospital reform (province)	−1.199^**^	−0.530^**^	0.073	−0.088^**^
	(−11.61)	(−13.72)	(1.20)	(−3.77)
Constant	4.819	1.207	−15.727^**^	−0.098
	(1.77)	(0.95)	(−5.50)	(−0.15)
Observations	68,553	52,894	52,487	68,954
Control	YES	YES	YES	YES
Year FE	YES	YES	YES	YES
Provcd FE	YES	YES	YES	YES

PS: Clustered individual-level standard errors in parentheses ^**^p < 0.01, ^*^p < 0.05.

## 4 Discussion

We used China’s public hospital reform, which was piloted in 2010 and fully rolled out in 2017, as a natural experiment to study the impact and microscopic mechanism of public hospital reform on the health inequality of Chinese residents. By utilizing the CFPS database, we matched the reform timelines of urban public hospitals in each province and accounted for the policy’s temporality. The calculated results indicate a 22.87% decrease in CI of health inequality before and after the reform. Using RIF to recenter the CI of health inequality and conducting an OLS panel regression, we found that the reform led to a 20.3% decrease in the Residents’ Inequality Index (RIF_CI). The paper proposes a tri-pathway chain mediation mechanism to explain the improvement in health inequality due to public hospital reform from a micro perspective of household consumption: (1) Public Hospital Reform – Family Medical Consumption – Health Inequality. Our research findings indicate that the reform, which abolished drug markups, significantly reduced household expenditures on purchasing medical services for daily needs. This finding differs somewhat from previous research conclusions discussed in the introduction (22–24). Differences in the time span, regions, and data sources of sample selection may account for the disparities in conclusions. The reduction in household medical expenses due to the reform lowering medicine prices has led to a decrease in household expenditures on purchasing medical services for daily needs, thereby enhancing the economic accessibility of medical service purchases for households and reducing the health inequality index faced by household members. (2) Public Hospital Reform – Healthy Leisure Consumption – Health Inequality. The overall reduction in the price of medical services alleviates precautionary savings motives induced by expenditure uncertainty and health uncertainty. This promotes consumer spending among residents (45), releasing budgets for other household expenditures. With the continuous improvement in the living standards of Chinese residents and the gradual increase in health awareness, the demand for health-related consumption has risen rapidly. The share of family healthy leisure consumption has also increased. For individuals with higher socioeconomic status, changes in medical expenses might not significantly impact household purchasing power or alter the household consumption structure. However, for ordinary families, the decrease in medical expenses not only expands total purchasing power but also reduces precautionary savings. This results in an increase in the budget for healthy leisure consumption, releasing demand for enjoyment-oriented consumption. In the absence of changes in existing medical services, the increased healthy leisure consumption contributes to enhancing individual health and reducing health disparities (46). (3) Public Hospital Reform – Family Medical Consumption – Family Healthy Leisure Consumption – Health Inequality. The increasingly diverse structure of household consumption is showing a trend of evolution from “basic” to “developmental” and “enjoyment” consumption upgrades (47). Therefore, the decrease in household medical expenses will also directly lead to an increase in leisure health consumption.

In the aforementioned indirect effects, the contribution of the increase in leisure health consumption is relatively larger. This conclusion is similar to previous research on the relationship between hierarchical health consumption and health inequality. For example, a study conducted using data from the 2015 survey on the living conditions of residents in large cities analyzed the actual effects of the new healthcare system reform on health economics. They concluded that health status is mainly determined by two aspects of health economic behavior: medical expenditure and leisure health consumption. Leisure health consumption is more likely than medical expenditure to be an effective pathway to promote health (46). However, the authors did not explore the chained relationship between household medical expenditure and leisure health consumption in relation to reform and health inequality. And we have enhanced this part of the research.

Additionally, we further demonstrated that the impact of reform on health inequality varies with education levels and regional population structures. In the rapidly evolving digital economy and the catalysis of the COVID-19 pandemic, the integration of healthcare and the internet has formed a unified service system encompassing both online and offline medical services. Public hospital reform, facilitated by telemedicine and initiatives such as the establishment of internet hospitals and provision of online diagnostic services, employs digital means to deliver high-quality medical services to the public. However, the effectiveness of the process from the improvement in healthcare service supply to the enhancement of residents’ access to health resources depends on residents’ abilities to access, assess, and utilize medical information. These abilities are typically determined by education levels. Empirical results (Table 6A) validate our hypothesis. The different demographic structures also affect the impact of public hospital reform on the health inequality index. Household medical service expenditures are expected to significantly increase with the trend of population aging (48). Public hospital reform can benefit families in regions with a high degree of aging. Moreover, studies suggest that older adults have significant potential demand in areas such as education, culture, entertainment, and health products (49). The increase in healthy leisure activities, such as tourism, has a significant impact on the physical and mental health of older adults (50). But the research results (Table 6B) do not align with our discussion. In regions with a severe aging population, public hospital reform does not effectively suppress health inequality. Possible reasons include the fact that, although the older adult are the main consumers of health, their willingness for pleasure-oriented consumption is limited, and they prioritize preventive savings against risks. Additionally, the development of the health industry is still in its early stages, limiting its ability to provide high-quality health products and services, which, in turn, constrains the demand for health consumption.

As far as we know, this is the latest interpretation of the policy effects of China’s public hospital reform on improving residents’ health inequality using household data from 2012 to 2020, along with the micro-level impact pathways. This finding suggests that the healthcare reform measures, which reduce out-of-pocket medical expenses for residents and enhance the rational allocation of medical resources, are a correct and feasible direction for reform, with a positive impact on reducing the residents’ health inequality index. Furthermore, this study decomposes family health consumption into medical expenditure and healthy leisure consumption. The process of transforming the budget for medical expenses into a budget for healthy leisure expenses is defined as the upgrading of the family health consumption structure. The study uses a chain mediation model to verify the micro-pathway of “public hospital reform – upgrading family health consumption – reducing health inequality.” Finally, we examines the changes in the coefficient $\alpha_{1}$ in different education groups and regions with varying degrees of aging. It finds that basic education is one of the thresholds for Chinese residents to gain opportunities for health equity from the reform. Meanwhile, the excessive preventive savings mindset and the insufficient development of the health industry limit the ability of the older adult population to obtain health equity from public hospital reform measures. Based on this, the paper suggests that reform policies in the healthcare sector should be combined with policies to promote universal basic education, develop the silver economy, and drive the health industry. Only through coordinated efforts can better results be achieved. This provides a theoretical basis for China to further deepen healthcare reform, strengthen multi-path collaboration, and enhance policy efforts to improve health opportunity inequality. It also offers experiential insights for other countries in formulating policies related to improving health inequality.

Our study has some limitations. Firstly, residents may underreport or misreport sensitive issues such as income and expenditure during the interviews, leading to errors in calculating the CI and RIF_CI of *per capita* net income and family consumption expenditure. However, the official data undergoes preliminary cleaning, and our large sample size helps mitigate some of these concerns. Secondly, both the CI and the RIF of health inequality are functions of residents’ health levels and income. This study uses self-assessed health scores of respondents in the CFPS as a measure of residents’ health levels. However, self-assessment scores are subjective evaluations and expectations of individuals about their health status (including physiological and psychological aspects), carrying the risk of overestimation or underestimation. Future research could consider using a combination of subjective and objective health indicators, such as the Quality of Well-Being scale (QWB), to reduce potential biases in the data. Thirdly, the empirical analysis lacks control for stable differences between provinces. After data cleaning, age range selection, and removal of samples with missing or outlier information, the remaining 121,447 valid samples are unevenly distributed among provinces and cities. Some provinces or cities have either no samples or only a few samples in a particular interview year. While our study does not directly compare differences between provinces or cities and uses various methods to demonstrate the robustness of the results, this issue could still introduce potential biases in the observed associations between the core explanatory variables and the dependent variables. Future research with a longer and more comprehensive dataset would help address this limitation.

## 5 Conclusion

In summary, our study concludes that the reform of China’s public hospitals has positively contributed to the improvement of residents’ health inequality, with one effective pathway being the alteration of the structure of family health consumption, resulting in additional health benefits for residents. Our findings also underscore the importance of universal higher education and the development of the health industry in the context of global digitization and the dual challenges of an aging population for addressing health inequality. We propose that reform policies in the medical field should integrate with policies promoting basic education, developing the silver economy, and advancing the health industry to work synergistically for better outcomes. To provide more targeted and effective strategies for local governments, future research could explore the evolving spatial patterns of health inequality in China and investigate the changes in key influencing factors.

## Data availability statement

The raw data supporting the conclusions of this article will be made available by the authors, without undue reservation.

## Author contributions

LJ: Writing – review & editing, Writing – original draft, Funding acquisition, Data curation, Conceptualization. YH: Writing – review & editing, Validation, Supervision, Funding acquisition, Conceptualization. CH: Writing – review & editing, Visualization, Data curation, Conceptualization.

## Footnotes

^1^In the CFPS data, there are instances where respondents answered “do not know,” “not applicable” or refused to answer certain questions. Official researchers assigned designated negative values to such cases in the database. Therefore, when processing the data, we removed data points where variables related to family medical, healthcare, cultural and recreational activities, travel, beauty expenses, family income, individual age, gender, self-rated health status, years of education, marital status, and urban–rural classification were less than zero. Additionally, we excluded all data points with missing values for core variables.

^2^Borrowing from existing research practices, the calculation process of the Concentration Index (CI) already incorporates information on income indicators. Therefore, income is a part of the health inequality index, and the control variables no longer include *per capita* income.
