# Supplementary material for: Public hospital reform, family health consumption and health inequality: evidence from China Family Panel Studies
Source: Front Public Health. 2024 Jun 18;12:1352417. doi: 10.3389/fpubh.2024.1352417 (PMC11217177; doi:10.3389/fpubh.2024.1352417)
Supplement: Supplementary file 1 [file Table_1.DOCX]

Table 1 The launch date of public hospital reform in some cities in 23 provinces and 4 municipalities in China can be queried

| Province/ Municipality | City | Time | Province/ Municipality | City | Time |
| --- | --- | --- | --- | --- | --- |
| Anhui | Anqing | 2015.04.01 | Hunan | Yueyang | 2016.10.01 |
|  | Hefei | 2015.04.01 |  | Xiangtan | 2016.10.01 |
|  | Xiancheng | 2015.04.01 |  | Hengyang | 2016.10.01 |
| Beijing | Beijing | 2017.04.08 |  | Yongzhou | 2016.10.01 |
| Chongqing | Chongqing | 2017.09.09 |  | Changsha | 2016.10.01 |
| Fujian | Ningde | 2015.06.20 |  | Loudi | 2016.10.01 |
|  | Putian | 2014.12.01 | Jiangsu | Wuxi | 2015.10.31 |
| Gansu | Qingyang | 2017.08.31 |  | Yangzhou | 2015.10.31 |
|  | Baiyin | 2017.08.31 |  | Lianyungang | 2015.10.31 |
|  | Lanzhou | 2017.08.31 | Jiangxi | Ji'an | 2017.09.09 |
|  | Pingliang | 2017.08.31 |  | Ganzhou | 2017.09.01 |
|  | Linxia | 2017.08.31 |  | Fuzhou | 2017.09.01 |
|  | Wuwei | 2017.08.31 | Jilin | Siping | 2017.08.26 |
|  | Tianshui | 2017.08.01 |  | Songyuan | 2016.12.01 |
|  | Longnan | 2017.08.01 |  | Tonghua | 2016.12.01 |
|  | Dingxi | 2017.08.01 | Liaoning | Tieling | 2017.8.26 |
| Guangdong | Guangzhou | 2017.07.15 |  | Fuxin | 2017.8.26 |
|  | Maoming | 2017.07.15 |  | Yingkou | 2017.8.26 |
|  | Yunfu | 2017.07.15 |  | Chaoyang | 2017.8.26 |
|  | Qingyuan | 2017.07.15 |  | Dalian | 2017.8.26 |
|  | Yangjiang | 2017.07.15 |  | Liaoyang | 2017.8.26 |
|  | Jieyang | 2017.07.06 |  | Huludao | 2017.8.26 |
|  | Shantou | 2017.07.01 |  | Dandong | 2017.8.26 |
|  | Meizhou | 2017.07.01 |  | Benxi | 2017.8.26 |
|  | Zhanjiang | 2017.06.19 |  | Shenyang | 2017.09.01 |
|  | Shaoguan | 2017.04.01 |  | Anshan | 2015.10.01 |
|  | Jiangmen | 2016.11.01 |  | Jinzhou | 2015.10.01 |
|  | Dongguan | 2015.10.15 | Shaanxi | Xi'an | 2017.09.01 |
|  | Zhuhai | 2015.03.29 |  | Huainan | 2017.04.01 |
| Guangxi | Wuzhou | 2017.03.09 |  | Yulin | 2017.04.01 |
|  | Guilin | 2016.12.10 | Shandong | Laiwu | 2016.10.28 |
|  | Fangchenggang | 2016.12.01 |  | Zibo | 2016.07.01 |
| Guizhou | Qiandongnan miao and dong | 2017.09.30 |  | Dezhou | 2016.07.01 |
|  | Qiannan buyei and miao | 2017.09.30 |  | Zaozhuang | 2016.06.31 |
|  | Qianxinan buyei and miao | 2017.09.30 |  | Yantai | 2016.06.30 |
|  | Zunyi | 2017.09.30 |  | Rizhao | 2016.06.01 |
| Hebei | Xiahuayuan | 2017.09.01 |  | Jinan | 2016.05.01 |
|  | Shijiazhuang | 2017.08.26 |  | Shanghai | 2017.02.01 |
|  | Qinhuangdao | 2017.08.26 | Shanxi | Lvliang | 2017.07.01 |
|  | Langfang | 2017.08.26 |  | Linfen | 2017.07.01 |
|  | Handan | 2017.07.04 |  | Datong | 2017.07.01 |
|  | Hengshui | 2016.12.01 |  | Xinzhou | 2017.07.01 |
|  | Cangzhou | 2016.11.30 |  | Changzhi | 2016.12.20 |
|  | Xingtai | 2016.11.26 |  | Yuncheng | 2016.11.01 |
| Heilongjiang | Jixi | 2017.08.01 |  | Taiyuan | 2016.11.01 |
|  | Harbin | 2017.08.01 | Sichuan | Chengdu | 2017.01.01 |
|  | Daxinganling | 2017.08.01 |  | Yibin | 2016.12.01 |
|  | Hegang | 2017.08.01 |  | Leshan | 2016.12.01 |
|  | Daqing | 2017.08.01 |  | Deyang | 2016.11.01 |
| Henan | Zhumadian | 2017.09.01 |  | Meishan | 2016.10.01 |
|  | Shangqiu | 2017.09.01 |  | Guangyuan | 2015.11.01 |
|  | Nanyang | 2017.09.01 |  | Liangshan yi | 2013.10.01 |
|  | Kaifeng | 2017.09.01 |  | Ganzi | 2013.10.01 |
|  | Xinxiang | 2017.09.01 | Tianjing | Tianjing | 2016.12.15 |
|  | Anyang | 2017.09.01 | Yunnan | Honghe hani and yi | 2017.06.01 |
|  | Xuchang | 2017.09.01 |  | Dali | 2017.06.01 |
|  | Luoyang | 2017.09.01 |  | Simao | 2017.06.01 |
|  | Pingdingshan | 2017.09.01 |  | Yuxi | 2015.12.01 |
|  | Zhengzhou | 2017.09.01 | Zhejiang | Hangzhou | 2014.04.01 |
|  | Zhoukou | 2017.09.01 |  | Taizhou | 2014.04.01 |
|  | Luohe | 2017.09.01 |  | Ningbo | 2013.12.23 |
|  | Xinyang | 2017.09.01 | Tianjing | Tianjing | 2016.12.15 |
|  | Jiaozuo | 2016.08.01 | Inner Mongoria |  | 2017.09.01 |
| Hubei | Yichang | 2017.07.31 | Ningxia |  | 2016.12.31 |
|  | Wuhan | 2017.01.01 |  | | |
|  | Xiangyang | 2016.09.12 |  |  |  |
| PS: 1.Cities in each province are listed in reverse chronological order of the implementation of the public hospital reforms. 2. Due to the unavailability of official notifications regarding the cancellation of drug markups in prefecture-level cities in Inner Mongolia Autonomous Region and Ningxia Hui Autonomous Region, we opted to directly use the time point when the entire province announced the cancellation of drug markups. | | | | | |

Table 2 Division of Eastern, Central and Western Provinces

| Area | Eastern | Central | Western |
| --- | --- | --- | --- |
| Province/ Municipality | Beijing | Henan | Gansu |
|  | Fujian | Heilongjiang | Guizhou |
|  | Guangdong | Hubei | Ningxia |
|  | Guangxi | Jiangxi | Shaanxi |
|  | Hainan | Anhui | Sichuan |
|  | Hebei | Hunan | Yunnan |
|  | Jiangsu | Jilin | Chongqing |
|  | Liaoning | Inner Mongoria IM | Qinghai |
|  | Shandong | Shanxi | Tibet |
|  | Shanghai |  | Xinjiang |
|  | Tianjing |  |  |
|  | Zhejiang |  |  |
